# The Effect of *Pseudomonas putida* on the Microbial Community in Casing Soil for the Cultivation of *Morchella sextelata*

**DOI:** 10.3390/jof11110775

**Published:** 2025-10-27

**Authors:** Ruifan Zou, Yuping Zhang, Lili Zhang, Ming Chen, Ling Xin, Lei Zhang

**Affiliations:** 1 Sericulture Research Institute, Anhui Academy of Agricultural Sciences, Hefei 230001, China; zouruifan0805@126.com (R.Z.); ypzhang6330@163.com (Y.Z.);; 2 Edible and Medicinal Mushroom Innovation Centre, Anhui Academy of Agricultural Sciences, Hefei 230001, China

**Keywords:** *Morchella*, *Pseudomonas putida*, microbial communities, 1-aminocyclopropane-1-carboxylic acid, microbial ecological networks

## Abstract

*Morels* are a rare edible and medicinal fungus. A major factor contributing to difficulties with their continuous cropping is alteration in soil microbial communities. *Pseudomonas putida* is a key microorganism in morel cultivation soils; it has garnered significant attention due to its ability to degrade 1-aminocyclopropane-1-carboxylic acid (ACC), a precursor of ethylene. However, the interaction between *Pseudomonas putida* and morels remains unclear. This study evaluated the growth-promoting potential of *P. putida* KT2440 by measuring the casing soil ACC content and assessing its ACC utilization capacity. Metagenomic sequencing was performed to assess the changes in soil microbial composition and function. The results indicated that ACC accumulated in the soil following morel cultivation and that *P*. *putida* KT2440 was capable of utilizing ACC as its sole nitrogen source for growth on plates. Inoculation enhanced the depletion of available nitrogen, phosphorus, and potassium; increased bacterial diversity; improved the stability of the soil microbial community; and caused the mycelium of morels to grow earlier. These processes occurred along with a decline in the abundance of the *Streptomyces* genus. Furthermore, a positive correlation was identified between the abundance of *P. putida* and ACC deaminase activity in the soil. Overall, this study examined the role of *Pseudomonas putida* inoculation in modulating the soil microbial community and metabolic processes within casing soil during *Morchella sextelata* cultivation. The findings indicate that *P. putida* inoculation promotes *Morchella* growth through ACC decomposition and microbial restructuring, offering a potential strategy for mitigating ethylene-related suppression in continuous cropping systems.

## 1. Introduction

*Morchella*, a highly valued edible and medicinal fungus classified within the phylum Ascomycota, the order Pezizales, and the family Morchellaceae [1], has garnered significant market interest due to its rich nutritional profile and substantial potential in health and pharmaceutical applications. As a highly nutritious food, the stipe and pileus of *Morchella sextelata* are equally rich in crude DF, various minerals and vitamins, several amino acids and 5′-nucleotides, total flavonoids, and ergothioneine [2]. Moreover, the polysaccharides extracted from it have been found to possess immunomodulatory and hepatoprotective capabilities [3,4]. Since the achievement of artificial cultivation using exogenous nutrient bags in 2012, the cultivation of morels in China has expanded to an area that reaches nearly 30,000 hectares over the last decade [5], covering over 20 provinces. However, this expansion has been accompanied by reduced yields and crop failure caused by continuous cropping-related obstacles [6].

These obstacles are alterations in soil physicochemical properties and shifts in the soil microbial community [7]. Continuous cropping of morels reduces the total potassium and available potassium in soil [8]. Therefore, farmers supplement potassium by adding wood ash to boost their mushroom yields [9]. On the other hand, continuous cropping also lowers the soil pH, which may cause the release of acidic substances during growth [10]. Microbial alpha diversity in continuously cropped soil is significantly lower than it is in soil used for the first year [11]. Current research suggests that casing soil harbors beneficial microorganisms that support the continuous cropping of edible fungi [12]. Casing cultivation is a critical step for enhancing *Morchella* yield and quality [13]. Casing soil provides thermal insulation, moisture retention, and nutrients for mycelial growth. More importantly, the diverse beneficial microorganisms within it supply essential substances for fungal growth, promote mycelial development, and stimulate fruiting body formation [14]. However, the specific functions and mechanisms of action of these microorganisms in *Morchella* casing soil remain incompletely understood and warrant further investigation. Commercial cultivation of edible fungi typically involves a series of controlled fermentation processes. Bacteria play crucial roles in substrate processing, suppressing fungal competitors and promoting mushroom development [15]. Other research highlights several beneficial effects of bacteria on edible fungi, including (1) secreting ligninases and cellulases to degrade complex polysaccharides, thereby providing nutrients [14]; (2) producing antibiotics to inhibit pathogenic infections [16]; and (3) consuming or breaking down growth-inhibiting substances, such as ethylene [17].

Ethylene, a gaseous phytohormone, regulates processes such as seed germination, root and stem growth, flowering, fruit ripening, organ senescence, and stress responses in plants [18]. However, it inhibits mycelial growth and fruiting body development in edible fungi [19]. In some species, ethylene is synthesized via the methionine metabolic pathway through ACC [20,21]. Certain microorganisms present in soil can decompose ACC by secreting ACC deaminase. They can decrease plant ethylene levels by catalyzing the cleavage of ACC to ammonia and α-ketobutyrate and increase plants’ resistance to a variety of environmental stresses [22,23]. Among them, *P. putida* has been widely reported [24]. In fungi, *P. putida* promotes the mycelial growth of *Agaricus bisporus* (the fourth most widely grown edible mushroom species) [25].

*P. putida*, a member of the *Pseudomonas* genus, is a widely reported plant-growth-promoting rhizobacterium. Chen et al. [20] identified a strain of *P. putida* producing ACC deaminase, which degrades ACC to reduce ethylene concentration, thereby promoting hyphal growth of *A. bisporus* and inducing primordium formation. Concurrently, the *Pseudomonas* species can biocontrol against edible mushroom pathogens. Certain members inhibit mycelial growth of Lecanicillium fungicola [20], while *P. putida* and *P. fluorescens* alleviate symptoms of brown blotch disease in *A. bisporus* [26].

Studies on bacterial communities in *Morchella* cultivation soils revealed that *Pedobacter*, *Pseudomonas*, *Stenotrophomonas*, and *Flavobacterium* constitute the core microbiome [27,28]. Significantly, *P. putida* was found to colonize *Morchella* hyphae and utilize fungal-derived carbon sources [29]. These findings suggest that *P. putida* may serve as a potential beneficial bacterium during *Morchella* development. However, it remains unclear whether *Morchella* synthesizes ethylene via the ACC pathway, or how *P. putida* inoculation affects microbial ecology and ethylene biosynthesis in *Morchella*.

Based on the literature cited above, we explored the role of *P. putida* as a bioinoculant that influences the casing-layer microbial community and promotes *Morchella* growth. Specifically, our objectives were to determine whether *P. putida* induces changes in the casing soil microbial community and whether its ACC deaminase activity promotes *Morchella* growth, ultimately optimizing the cultivation conditions. We conducted the following analyses: (1) determine the impact of *P. putida* inoculation on the diversity and structure of the *Morchella* casing-layer microbial community across different time periods; (2) identify species-specific effects of microorganisms on soil physicochemical properties following *P. putida* inoculation; and (3) assess the influence of *P. putida* inoculation on the ethylene metabolic pathways within the casing soil microorganisms.

## 2. Materials and Methods

### 2.1. Experimental Design

To characterize the effect of *P. putida* on the casing soil-associated microbial communities, we grew the most common *Morchella* species in China, *Morchella sextelata*, in Pindstrup substrate (0–6 mm for the cultivation substrate and the casing soil) under controlled conditions. The experimental greenhouse is located at the comprehensive experimental base of the Sericultural Research Institute of Anhui Academy of Agricultural Sciences in Hefei city, Anhui province (31.90° N, 117.21° E). The *P. putida* strain KT2440 was kindly provided by Professor Bai Zhonghu at Jiangnan University.

**Figure 1:**
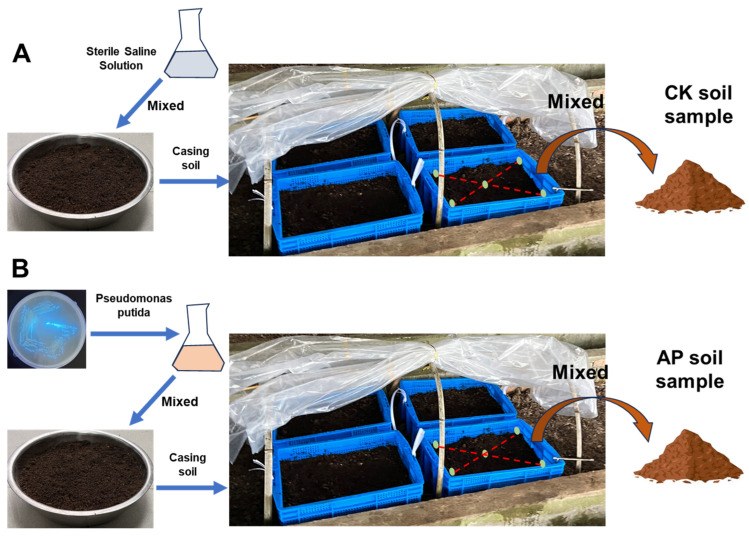
Experimental design overview of this study. Addition of (**A**) sterile saline (CK) and (**B**) *P. putida* inoculant groups (AP) with sampling and analysis at designated time points. After day 90 of experiment, number of *Morchella* fruiting bodies was counted once a week, and time of their emergence was noted.

In December 2024, *P. putida* KT2440 was cultured at 30 °C until it reached the logarithmic growth phase. Then, it was resuspended in sterile physiological saline to achieve a concentration of approximately 10^9^ CFU/mL. For the inoculation group, 50 mL of this bacterial suspension was mixed with 50 g of sterilized Pindstrup substrate to form an inoculant. Conversely, the control group was prepared by mixed 50 mL of sterile physiological saline with the same amount of substrate. Each planting tray was filled with 20 L of Pindstrup substrate. The *Morchella* spawn was crumbled into uniform fragments and evenly distributed over the tray surface. Subsequently, the casing soil was prepared by thoroughly mixed 5 L of sterilized Pindstrup substrate with either the inoculant or the control mixture. Each treatment group comprised four replicate pots. After planting, the substrate was watered thoroughly (Figure 1). Then, one nutrient bag (1 kg, 78% wheat + 20% corn cob + 1% lime + 1% gypsum) was placed 7–10 days post-sowing for each planting tray. Black PE film with holes was used to cover the pots to maintain soil moisture. We regularly checked the soil moisture and replenished water as needed. At the primordia formation stage, the substrate was thoroughly irrigated. The PE film mulch was subsequently removed and replaced with a 40 cm highly arched shelter covered with white polyethylene (PE) film. Ventilation holes were made in the covering to ensure adequate gas exchange. Throughout this period, the following key environmental parameters were maintained: air humidity > 85% and CO_2_ concentration < 2000 ppm. For each treatment group, four independent biological replicates were established, and all were subjected to soil properties analysis and DNA extraction. Statistical analyses were performed and graphical representations were created based on three randomly selected replicates from each group to ensure consistency and robustness.

### 2.2. Soil Sample Collection

To track the dynamic effects of *P. putida* inoculation on the microbial community throughout *Morchella* cultivation, we collected the soil samples at sequential stages (0 d, 30 d, 60 d, and 90 d) spanning from inoculation to mushroom emergence. The soil samples from each planting tray were collected within 0–5 cm of the casing soil. Within each tray, we took soil from a total of five points located at the four corners and the center, and these five soil samples were thoroughly mixed in the sampling bag to provide a composite soil sample [30,31]. Each soil sample was sealed in an aseptic Ziplock bag. After collection, the fresh soil samples were placed on ice and immediately transported back to the laboratory, where each composite soil sample was lightly sieved (<3 mm) to homogenize the soil and to remove some stones, larger particles, and soil organisms [32]. These samples were divided into two aliquots; one was preserved at −80 °C for subsequent metagenomic sequencing, and the other was air-dried for determining basic soil physicochemical indices, including pH.

### 2.3. Strains and Cultivation Methods

*M. sextelata* was inoculated onto the center of a potato dextrose agar plate (PDA, Solarbio, Beijing, China), incubated at 22 °C for 5 days, and then transferred to a 1 kg sterile cultivation bag (90% wheat and 10% corn cob) for about 30 days. After the mycelium fully colonized the surface, the substrate was used for the above-described experiments. *P. putida* KT2440 was streaked onto LB (Solarbio, Beijing, China) agar plates and incubated at 30 °C for 3 days. A single colony was then picked and cultured in LB liquid medium at 30 °C and 200 rpm and was used for the experiments.

### 2.4. Detection of ACC Content and ACC Utilization Ability

The determination of ACC content in soil was performed using high-performance liquid chromatography (HPLC). Specifically, 2 g of soil was ground in liquid nitrogen, and then subjected to ultrasonic treatment in deionized water for 30 min. The mixture was centrifuged at 10,000 r/min for 5 min at 4 °C. The supernatant was collected, adjusted to pH 4.0, and mixed with chloroform. After another centrifugation step under the same conditions, the supernatant was passed through an MCX column. The eluate was then analyzed by HPLC-MS/MS using an ACC standard solution as a reference.

DF complete and ADF Culture medium (with ACC as the sole nitrogen source) [33] plates were prepared. The tested bacterial strains *Escherichia coli* DH5α and *P. putida* KT2440 were streak-plated and incubated at 30 °C for 5 days. Growth was observed, and fluorescence capability was verified under UV illumination at a wavelength of 366 nm.

### 2.5. Soil Physicochemical Properties

The basic soil chemical properties were analyzed using the following methods. Soil pH was measured in a 1:2.5 (*w*/*v*) soil–water suspension using the glass electrode method according to NY/T 1121.2-2006 [34]. The amount of soil organic carbon (SOC) was determined by the potassium dichromate oxidation–external heating method (NY/T 1121.6-2006) [34]. The total nitrogen (TN) was measured using the Kjeldahl method, following sulfuric acid digestion with a catalyst (LY/T 1228-2015) [35]. The total phosphorus (TP) was analyzed by the molybdenum-antimony anti-spectrophotometric method after sodium hydroxide fusion (GB/T 9837-1988) [36]. The total potassium (TK) was determined by flame photometry after sodium hydroxide fusion (NY/T 87-1988) [37]. Available nitrogen (AN) was assessed using the alkaline hydrolysis diffusion method (LY/T 1228-2015) [38]. Available phosphorus (AvP) was extracted with ammonium fluoride–hydrochloric acid solution and measured by molybdenum-antimony anti-colorimetry (NY/T 1121.7-2014) [39]. Available potassium (AK) was extracted with ammonium acetate and determined via flame photometry (NY/T 889-2004) [39].

### 2.6. DNA Extraction

A total of 0.3 g of casing soil sample was used to extract total genomic DNA with the PowerSoil DNA Isolation Kit (MO BIO Laboratories, Carlsbad, CA, USA) according to the manufacturer’s instructions. Concentration and purity of extracted DNA were determined with NanoDrop 2000 (NanoDrop Technologies Inc., Wilmington, DE, USA). DNA quality was checked using 1% agarose gel.

### 2.7. Metagenomic Sequencing and Data Processing

The DNA samples were delivered to Majorbio Bio-pharm Technology Co., Ltd. (Shanghai, China) for further metagenomics sequencing. The sequencing library was constructed using the Illumina NovaSeq 6000 platform (Illumina Inc., San Diego, CA, USA) using paired ends (2  ×  150 bp) with the NovaSeq X Series 25B Reagent Kit according to the manufacturer’s instructions (www.illumina.com, accessed on 19 May 2025). The data were analyzed on the Majorbio Cloud Platform. Briefly, the paired-end Illumina reads were trimmed of adaptors, and low-quality reads (length < 50 bp or with a quality value < 20) were removed by fastp (https://github.com/OpenGene/fastp, accessed on 21 May 2025, version 0.23.0). The reads that contained adapters, low-quality bases, and 10% undefined bases were removed. A total of ~154 Gb clean data was obtained from 32 samples. MEGAHIT (version 1.1.2) software was used to assemble high-quality reads, and contigs that were shorter than 300 bp were discarded. After gene prediction with Prodigal v2.6.3, the genes were clustered to remove redundant sequences using CD-Hit v4.6.1 at 90% identity and 90% coverage. The metagenomic sequencing data associated with this project were deposited in the NCBI Short Read Archive database (Accession Number: PRJNA1311151).

### 2.8. Taxonomic and Functional Annotation

These non-redundant genes were annotated for function and classification by aligning them against the NCBI NR database using DIAMOND (version 2.0.13) with an E-value threshold of 1 × 10^−5^. For signaling pathway enrichment analysis, the KEGG pathway database was employed. We used the Kruskal–Wallis rank-sum test, one-way analysis of variance (ANOVA), and LSD (*p* < 0.05) for multiple comparisons of the differences in soil properties. We annotated the fungal and bacterial communities separately using NR for alpha and beta diversity analyses. Alpha diversity was calculated based on the algorithm for index analysis provided by mothur (version v.1.30.2, https://mothur.org/wiki/calculators/, accessed on 29 May 2025) using R (version 3.3.1). Beta diversity was analyzed and plotted using R language (version 3.3.1) for PCoA statistics, and the distance algorithm was Bray–Curtis. The neutral community model (NCM) used the R language software packages Hmisc (version 5.0.1), minpack.lm (version 1.2.3), and getopt (version 1.20.3) for analysis to visualize the community structure changes. Redundancy (RDA uses the rda analysis and plotting functions in the vegan package of R version 2.4.3) and mantel test heatmap (uses the R language version 3.3.1 and the vegan package version 2.4.3) analyses were performed to elucidate the relationships between the microbial communities and the soil physicochemical factors. We used LEfSe (one-against-all) to analyze the intergroup differences in species distribution across different periods and employed LDA discriminant analysis to estimate how the differential species affect component differentiation. Based on taxonomic and functional annotations and the abundance profile of non-redundant genes, differential analyses were carried out at the taxonomic, functional, and gene-wise levels using Kruskal–Wallis test.

## 3. Results

### 3.1. Changes in ACC Content and P. putida ACC Utilization Capacity

**Figure 2:**
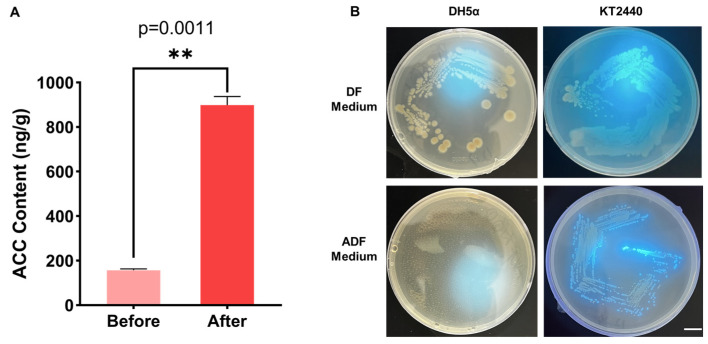
Casing soil ACC concentration and ACC utilization ability of KT2440. (**A**) Concentration of ACC in soil before and after planting morels. *n* = 3. ** *p* value < 0.01. (**B**) Growth conditions of *E. coli* DH5α and *P. putida* KT2440 on DF and ADF medium. We verified fluorescence capability using ultraviolet light with wavelength of 366 nm.

The ACC content in the casing soil was measured before and after *Morchella* cultivation by HPLC-MS/MS (Figure S1). Analysis of the data reveals that the ACC levels were significantly higher after cultivation compared to those of the unplanted fresh soil (Figure 2A), suggesting that *Morchella* may possess an ACC-dependent ethylene biosynthesis pathway.

To assess the ACC utilization capacity of *P. putida* KT2440, the strain was cultured on DF and ADF medium for 7 days. Figure 2B demonstrates that although the growth on the ADF plates was inferior to that on the DF medium, it was significantly better than that of *E. coli* DH5α, which did not grow. These results indicate that *P. putida* KT2440 can utilize ACC as a sole nitrogen source.

### 3.2. Changes in Soil Physicochemical Properties

**Figure 3:**
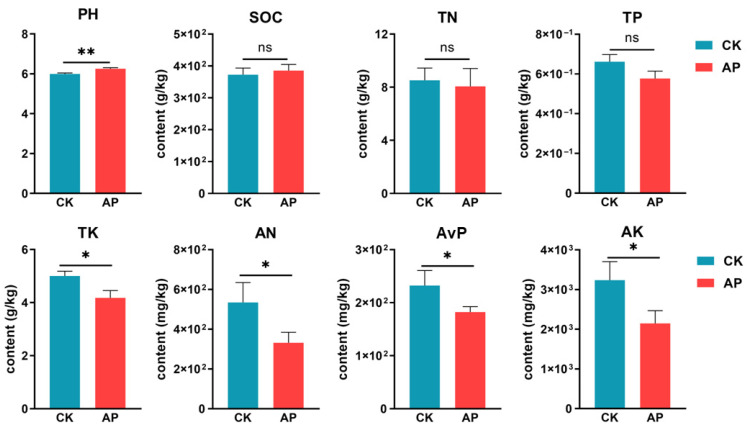
Effects of *P. putida* inoculation on casing soil physicochemical properties. PH, hydrogen ion concentration; SOC, soil organic carbon; TN, total nitrogen; TP, total phosphorus; TK, total potassium; AN, available nitrogen; AvP, available phosphorus; AK available potassium. Significant differences between CK and AP treatments are indicated by *. *n* = 4. ns means no significant difference * *p* value < 0.05, ** *p* value < 0.01.

The casing soil samples collected at 90 days post-cultivation were analyzed. Compared to the control group (Figure 3), the soil pH significantly increased following *P. putida* inoculation (*p* < 0.01). Conversely, significant decreases were observed in the AP group for TK, AN, AvP, and AK (*p* < 0.05). Although the control group showed higher total phosphorus (TP) values than the inoculated group, this difference was not statistically significant.

### 3.3. Alterations in Microbial Diversity of Casing Soil Following P. putida Inoculation

**Figure 4:**
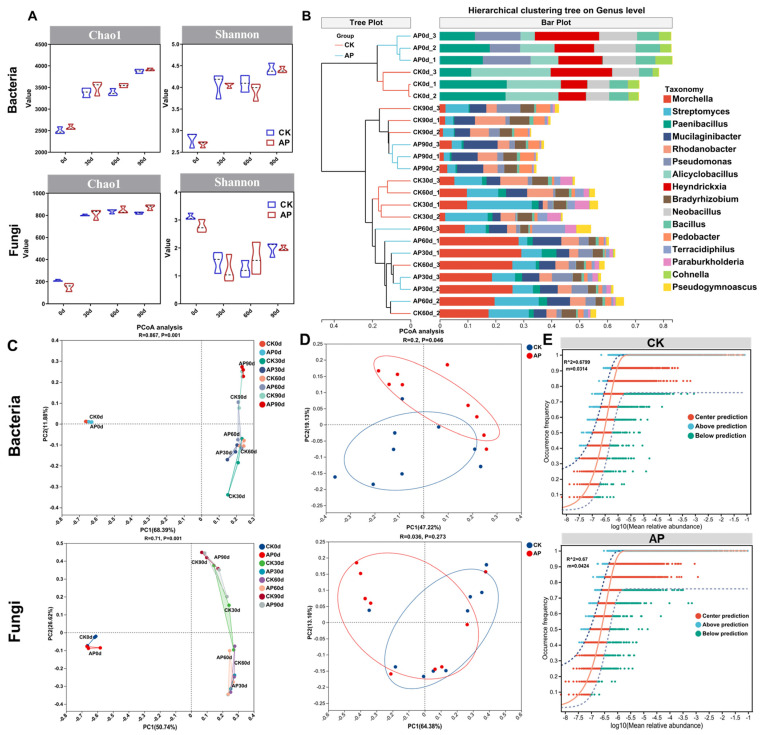
Effects of *P. putida* inoculation on casing soil microbial diversity. (**A**) Bacterial and fungal alpha diversity indices of casing soils at 0 d, 30 d, 60 d, and 90 d. (**B**) Hierarchical clustering tree of microbial community at genus level in different samples. Number represents three biological replicates for each sample. (**C**) PCoA analysis of bacteria and fungi in CK and AP groups was conducted at 0, 30, 60, and 90 days. Significant difference between 0 days and 30–90 days. *n* = 3. (**D**) PCoA analysis of bacteria and fungi in CK and AP group at 30–90 days. *n* = 3. The blue circles represent the control group, and the red circles represent the inoculation group. (**E**) Fitting of neutral community model (NCM) for the assembly of microbial communities in AP group compared to CK group. R^2^ indicates overall goodness of fit for this model; m represents migration rate at community level.

For the bacterial communities, the Chao index increased significantly over time (*p* < 0.001; see Table S1), with the values at 90 days being significantly higher than those at 30 and 60 days, which were in turn higher than the baseline (0 day). Notably, no significant differences were observed between the control (CK) and inoculated (AP) groups at any time point (Figure 4A), indicating that *P. putida* inoculation did not alter the bacterial richness. The Shannon index also increased over time. In the CK group, the values at 30, 60, and 90 days were significantly higher than those at the baseline (*p* < 0.001), with further differences between 90 days and the earlier time points (*p* < 0.001). The AP group showed no such temporal variation (see Table S1). Although no significant differences were detected between the CK and AP groups at any time point (Figure 4A), these findings suggest bacterial diversity fluctuations were more subdued in inoculated soil.

For the fungal communities, the Chao index remained stable from 30 to 90 days in both the CK and AP groups, with the values being significantly higher than those at 0 days (*p* < 0.001). No significant differences were observed between the CK and AP groups across all the periods (Figure 4A). Conversely, the Shannon index peaked at 0 days, and subsequently declined sharply (*p* < 0.001). Fungal diversity stabilized from 30 to 90 days in the AP group, while the CK group exhibited an initial drop, followed by a rebound (see Table S1), indicating a significant reduction in fungal diversity over time. Despite the absence of intergroup differences, the AP group demonstrated greater stability in fungal community richness.

Hierarchical cluster analysis was performed on 24 samples across eight groups (Figure 4B). The 0-day samples from the CK and AP groups clustered separately on the same branch, with Pseudomonas being the only species showing distinct differences. The remaining 30- to 90-day samples clustered together on a single branch. Within this branch, the 90-day samples from both groups clustered separately on the same branch, while the 30- and 60-day samples from each group exhibited some species similarity and clustered together.

Principal coordinate analysis (PCoA) revealed distinct differences in soil microbial composition between day 0 and later time points (30–90 days) (Figure 4C); this indicates that the microbial diversity in sterilized soil significantly increased after the cultivation of morel mushrooms. Exclusion of the 0-day data showed significant divergence in bacterial communities between the AP and CK groups from 30 to 90 days (*p* < 0.05), though no such difference was observed for the fungi (Figure 4D).

The assembly dynamics of microbial communities in the AP and CK groups were analyzed employing the neutral community model (Figure 4E). A higher R^2^ value indicates closer alignment of the observed community structure with the neutral model, implying greater stochasticity in assembly. The m parameter reflects the community size and species pool richness. The inclusion of *P. putida* improved the model’s ability to account for species diversity distribution, as evidenced by a higher m value. This suggests stronger species migration dynamics in the AP group compared to the CK group, which displayed lower migration rates.

### 3.4. Alterations in Microbial Composition of Casing Soil Induced by P. putida Inoculation

**Figure 5:**
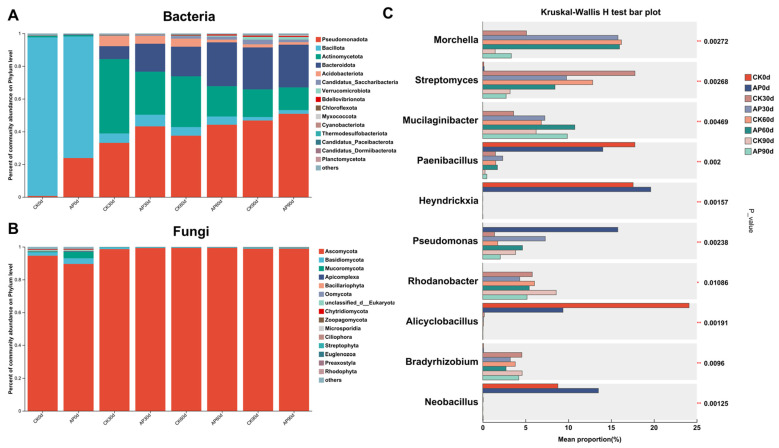
Dominant microorganisms in CK and AP groups at different times. Bacteria (**A**) and fungi (**B**) dominant microbial phyla (top fifteen by relative abundance). (**C**) Multiple group comparisons at microbial genus level (top ten by relative abundance). * *p* value < 0.05, ** *p* value < 0.01.

The phylum-level analysis revealed Bacillota as the predominant bacterial phylum at day 0 (Figure 5A), accounting for over 70% in both the groups. Following *P. putida* inoculation, the relative abundance of Pseudomonadota progressively increased, with the inoculated group exhibiting higher proportions (23.8%, 43.2%, 44.2%, and 50.9%) compared to the control group (0.6%, 33.1%, 37.5%, and 46.8%). Additionally, Bacteroidota and Actinobacteriota displayed relatively high abundances between days 30 and 90. The relative abundance of Bacteroidota increased over time, with lower levels in the control group (7.9%, 18.1%, and 25.6%) than in the inoculated group (17.1%, 26.8%, and 26.1%). Conversely, Actinobacteriota abundance declined over time, with the control group consistently exhibiting higher relative abundances (45.4%, 31.0%, an d16.9%) than the inoculated group (26.3%, 18.5%, and 13.9%). The predominant fungi were Ascomycota (Figure 5B). Except for a small amount of other phyla fungi in the CK and AP groups at 0 d, the proportion of Ascomycota in the other groups was above 98%.

The genus-level analysis of the top 10 microbial taxa (determined by the Kruskal–Wallis test; Figure 5C) showed that the relative abundance of *Morchella* initially increased before decreasing. The inoculated group exhibited higher *Morchella* abundance than the control group at days 30 and 90. Similarly, *Pseudomonas* abundance in the inoculated group surpassed that of the control group at days 0, 30, and 60; *Streptomyces* and *Bradyrhizobium* abundance was significantly lower than that of the control group at 30–60 days; and that of Mucilaginibacter was significantly higher in the control group than in the inoculated group over a 30-to90-day period. The four genera Paenibacillus, Heyndrickxia, Alicyclobacillus, and Neobacillus exhibited higher proportions immediately after inoculation following 0 day of sterilization, but their proportions decreased significantly to near zero between 30 and 90 days.

### 3.5. Alteration of Microbial Functions in Casing Soil Following Pseudomonas putida Inoculation

**Figure 6:**
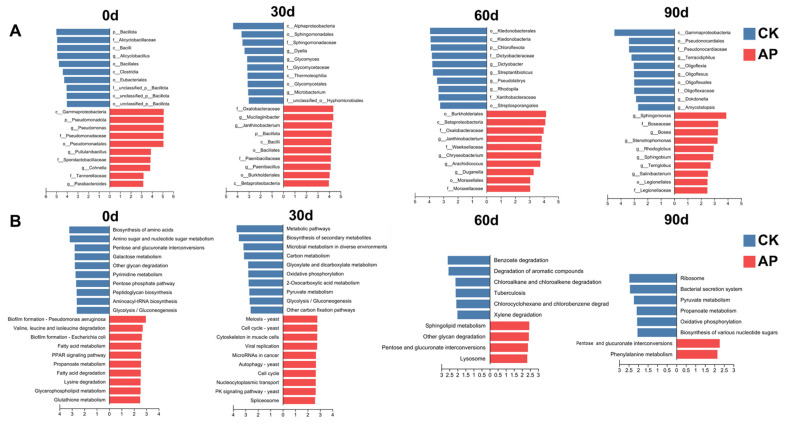
Linear discriminant analysis effect size (LEfSe) analysis at 0 d, 30 d, 60 d, and 90 d. (**A**) Top 10 species with greatest difference in microbial abundance between CK and AP groups. Linear discriminant analysis score ≥ 2.5. (**B**) Top 10 differentially expressed species in CK and AP groups in KEGG pathways (level 3). Linear discriminant analysis score ≥ 2.0.

The LEfSe analysis revealed significant temporal shifts in both bacterial taxonomy and KEGG pathway enrichment between the *Pseudomonas putida*-inoculated (AP) and control (CK) groups throughout the 90-day experiment (Figure 6A,B). At 0 day, prior to any treatment effect, the bacterial community in the AP group was already characterized by a higher abundance of the phylum Bacillota (Alicyclobacillus). In contrast, the CK group was enriched in Gammaproteobacteria, including the genus Pseudomonas. Functionally, the AP group showed enrichment in pathways for biofilm formation and fatty acid degradation, while the CK group was enriched in biosynthetic pathways like biosynthesis of amino acids and amino sugar metabolism. By 30 days, the inoculated *P. putida* had significantly altered the community structure. The AP group became enriched with Alphaproteobacteria (Sphingomonadaceae) and Bacillota (Paenibacillus), whereas the CK group was dominated by Betaproteobacteria (Oxalobacteraceae) and Bacilli. This was accompanied by a clear functional divergence; the AP group shifted toward central carbon metabolism (pyruvate metabolism and oxidative phosphorylation), while the CK group was enriched in eukaryotic-associated pathways, such as the cell cycle and meiosis (yeast). At 60 days, the taxonomic differences intensified. The AP group was enriched with Chloroflexota (Kiedonobacterales) and Actinobacteria (Streptosporangiales), while the CK group was characterized by Betaproteobacteria (Burkholderiales) and Bacteroidota (Chryseobacterium). Functionally, the AP group exhibited a strong capacity for degradation of aromatic compounds (benzoate and xylene), contrasting with the CK group’s enrichment in sphingolipid metabolism. At 90 days, the AP group’s community was dominated by Gammaproteobacteria and Actinobacteria (Pseudonocardiales), including genera like Amycolatopsis. The CK group was enriched with Alphaproteobacteria (Sphingomonas) and Bacteroidota (Salinibacterium). Metagenomic functions in the AP group were related to bacterial secretion systems and energy metabolism, whereas the CK group showed enrichment in the ribosome pathway.

### 3.6. Pseudomonas putida Inoculation Alters Interactions Between Environmental Factors and Microbial Communities

**Figure 7:**
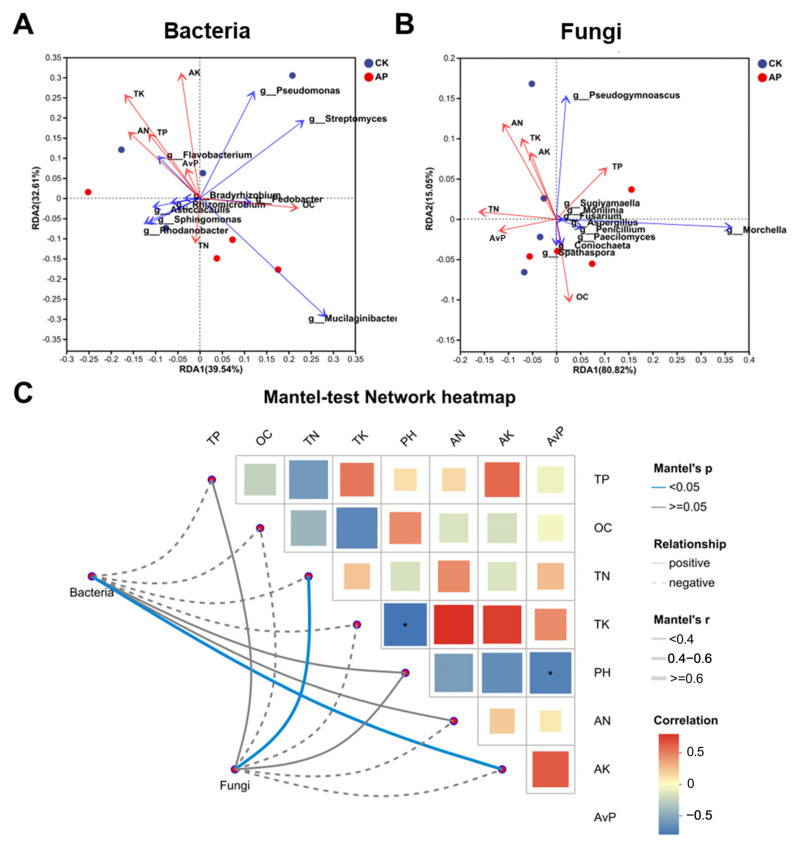
Variations in bacteria (**A**) and fungi community composition (**B**) associated with significant environmental factors are shown as RDA biplots, respectively. (**C**) Relationships among bacterial and fungi community composition and environmental variables in casing soil. * *p* value < 0.05.

Redundancy analysis (RDA) investigated the dependence of microbial community composition on environmental factors; the blue arrows indicate the top ten species by abundance. For the bacterial communities (Figure 7A), the first and second ordination axes explained 39.54% and 32.61% of the variation, respectively (cumulative 72.15%). AK, AN, AvP, TK, and TP were positively correlated with *P. putida*; the correlation between the other bacteria and the soil physicochemical properties is shown in the RDA analysis comparison table (Table S2). For the fungal communities (Figure 7B), the first and second ordination axes accounted for 80.82% and 15.05% of the variation, respectively (cumulative 95.87%). AN and TK showed the strongest correlations with fungal species distribution, while TP and OC were positively correlated with *Morchella*. The correlation analyses and the Mantel tests (Figure 7C) indicated the bacterial community composition was significantly positively correlated with AK (*p* < 0.05), while the fungal community composition was significantly positively correlated with TN (*p* < 0.05). Conversely, pH was significantly negatively correlated with AvP and TK (*p* < 0.05).

### 3.7. Effects of Pseudomonas putida Inoculation on KEGG- and ACC-Related Metabolic Pathways

**Figure 8:**
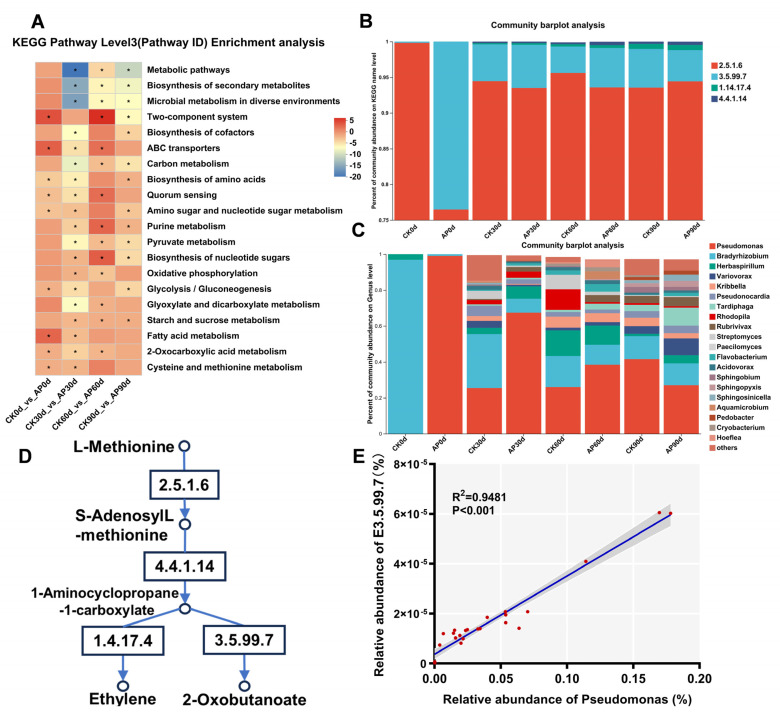
Effects of inoculating *P. putida* on microbial functions. (**A**) KEGG pathway enrichment analysis in top 20 enriched pathways. Microbial gene functions were changed after inoculating *P. putida*. Blue, enriched in CK group; red, enriched in AP group. Asterisk denotes reporter score of pathways  >  1.65 or  <−1.65. * *p* value < 0.05. (**B**) KEGG name levels of these several enzymes during four periods of CK and AP groups. S-adenosylmethionine synthetase (2.5.1.6), 1-aminocyclopropane-1-carboxylate synthase (4.4.1.14), aminocyclopropanecarboxylate oxidase (1.14.17.4), and 1-aminocyclopropane-1-carboxylate deaminase (3.5.99.7) (**C**) Relative abundance map of species producing ACC deaminase. (**D**) Metabolic pathway of L-methionine resulted in ethylene and 2-Oxobutanoate. (**E**) Correlation between *Pseudomonas* and enzyme 3.5.99.7.

The KEGG pathway enrichment analysis revealed distinct functional profiles between the inoculated (AP) and control (CK) groups in both the top 20 enriched pathways (Figure 8A) and in carbon, nitrogen, and phosphorus cycling (Figure S2). The control group was significantly enriched in fundamental microbial metabolic and biosynthetic pathways, such as metabolic pathways (ko01100), biosynthesis of secondary metabolites (ko01110), microbial metabolism in diverse environments (ko01120), carbon metabolism (ko01200), and degradation of aromatic compounds (ko01220). In contrast, the inoculated group at 60 days displayed a distinct functional emphasis, with primary enrichments in the two-component system (ko02020), nucleotide sugar biosynthesis (ko01250), and purine metabolism (ko00230).

Based on the methionine-to-ethylene metabolic pathway (M00368), a schematic diagram illustrates the metabolic route of L-methionine via S-adenosylmethionine synthetase (2.5.1.6), 1-aminocyclopropane-1-carboxylate synthase (4.4.1.14), aminocyclopropanecarboxylate oxidase (1.14.17.4), and 1-aminocyclopropane-1-carboxylate deaminase (3.5.99.7), ultimately leading to ACC synthesis (Figure 8D). Comparing abundance at the KEGG name level using a gene set for this pathway revealed that 2.5.1.6 exhibited the highest abundance across all the time periods. The second most abundant enzyme, 3.5.99.7, peaked at day 0 post-inoculation. Additionally, the average abundance of 3.5.99.7 in the inoculated groups was higher than that in the control groups at 30 and 60 days (Figure 8B). We constructed a gene set for ACC deaminase (K01505) for NR species annotation and generated species abundance maps (Figure 8C). Pseudomonas consistently ranked among the top species in abundance for ACC deaminase synthesis. Within the AP group, the relative abundance of Pseudomonas gradually declined from an initial level exceeding 99% to below 30% at 90 days. Conversely, in the CK group, its proportion gradually increased from 0% to over 40%, surpassing that of the AP group. Beyond Pseudomonas, the other significant contributors within the indigenous microbial community included *Bradyrhizobium* and *Herbaspirillum*. To assess the influence of *Pseudomonas* addition to the casing on the abundance of enzyme 3.5.99.7, regression analysis was conducted on the relative abundance within both the AP and CK communities. This analysis revealed that the abundance of enzyme 3.5.99.7 showed a positive correlation (R^2^ = 0.9481, *p* < 0.001) with a relative abundance of *Pseudomonas* throughout the cultivation period (Figure 8E).

## 4. Discussion

### 4.1. Morels Accumulate Ethylene During Their Growth Process

Ethylene is an auto-inhibitory substance in the growth of edible mushrooms; elevated ethylene levels can inhibit mycelial expansion and even suppress the formation of fruiting bodies. In most microorganisms, ethylene is synthesized through either the KMBA pathway, in which methionine is metabolized to 2-keto-4-methylthiobutyric acid, or the EFE pathway, which leads to the production of 2-ketoglutaric acid. However, in the soil-dependent cultivation of *A*. *bisporus*, ethylene is produced via the ACC pathway. In this study, ACC accumulation was detected in the substrate following *M. sextelata* cultivation (Figure 2A), suggesting that morels may also synthesize ethylene through the ACC pathway.

*P. putida* is a beneficial bacterium in plant rhizosphere ecosystems, known to alleviate drought stress and enhance disease resistance [40,41]. It is a core microbial species in the soil surrounding morel fruiting bodies [42]. Research indicates that morels “cultivate” *P. putida* by secreting metabolites that promote bacterial growth, while also absorbing and utilizing the bacteria as a nutrient source [29]. When grown in an ADF medium with ACC as the sole nitrogen source, *P*. *putida* KT2440 showed normal growth (Figure 2B), indicating that this strain has the potential to mitigate continuous cropping obstacles in morels through ACC degradation.

### 4.2. P. putida Affected the Microbial Composition in the Casing Soil

In edible mushroom cultivation, certain species require specific symbiotic fungi for the development of fruiting bodies; otherwise, the yields remain low, or fruiting may not occur at all. For example, *Tremella fuciformis* depends on *Hypoxylon stygium* as its symbiotic partner [43], while *Tremella aurantia* relies on *Stereum hirsutum* [44]. Additionally, some edible fungi—such as *Phlebopus portentosus* [45], *Dictyophora* [46], *Ganoderma* [47,48], and *Stropharia rugosoannulata* [49,50]—require casing soil for normal fruiting. Casing soil not only offers mechanical support for the development of fruiting bodies, but also improves the physicochemical properties of the substrate, including water retention, aeration, temperature regulation, and nutrient availability. Importantly, microorganisms present in the covering material can play a significant role in promoting mycelial growth and fruiting, as documented in *A. bisporus* and *Morchella* [15,51].

In this study, since the substrate was uniformly sterilized prior to planting, both bacterial and fungal richness at day 0 were significantly lower than in the three subsequent sampling periods (Figure 4). After morel cultivation, both the abundance and diversity of bacteria increased. For the fungi, although the species richness increased, the overall diversity decreased markedly. This shift was characterized by the dominance of the phylum Ascomycota, primarily represented by morel fungi (Figure 5B). Consistent with the life cycle of morels, extensive mycelial mats formed on the surface of the soil cover after inoculation [52]. This proliferation suppressed the growth of other fungi, thereby reducing fungal diversity in the soil. At 30 days post-inoculation, the abundance of morel mycelium in the inoculated group reached its peak (Figure 5C), whereas the peak occurred at 60 days in the control group. This indicates that inoculation with *P. putida* accelerated the mycelial growth process of *M. sextelata*. Morels’ mycelium growth aids in the assimilation, absorption, and storage of nutrients, and then it undergoes reproductive growth and develops into ascocarps under appropriate environmental conditions [53]. Accelerated mycelial colonization of the surface facilitates faster nutrient uptake and accumulation, thereby promoting morel mushroom growth.

The microbial communities assembled during the distinct growth stages of edible fungi vary significantly, with each assemblage performing unique functional roles. In *A. bisporus*, as the mushroom cropping process progressed, and relative abundance of Actinobacteria and Bacillota increased successively after both the first and second mushroom flushes. Relative abundance of Pseumonadota decreased after the first mushroom flush, while relative abundance of Bacteroidota increased between the first and second flushes, but decreased between the second flush and third flush [15,54,55]. Under the treatment of *P. putida* in this study, these two phyla, which are also commonly found in disease-suppressing and high-fertility soils, jointly dominate and form a powerful, mutually beneficial functional network, may play a crucial role in driving the mycelial proliferation and primordium formation of morels.

In this study, at the phylum level, sterilization proved devastating to the original microbial community, leaving the Bacillota phylum as the dominant microbial group This dominance stems from the high thermal tolerance exhibited by most Bacillota members, as evidenced by their high abundance in post-fire soils [56]. The initial dominance followed by gradual decline in both Actinomycota and Bacillota abundance may be attributed to intense competition and interactions among microbial species. At the genus level, the inoculation of *P. putida* resulted in increased levels of *Bacteroidota* and *Pseudomonadota* in the soil, while that of *Streptomyces* (belonging to *Actinomycetota*) decreased. Over time, the inoculated group exhibited a sequential microbial enrichment pattern, from γ-Proteobacteria to β-Proteobacteria (*Burkholderiales* and *Oxalobacteraceae*), and finally to α-Proteobacteria (*Stenotrophomonas* and *Legionellales*). In contrast, the control (CK) group transitioned from Bacillota to α-Proteobacteria (*Sphingomonadales*), and then to Chloroflexota, and ultimately to Actinobacteria (*Pseudonocardiales* and *Amycolatopsis*). During the first 30 days, both the AP (inoculated) and CK groups were dominated by α-Proteobacteria, which are capable of degrading simple organic compounds. Notably, by day 30, the CK group already hosted *Sphingomonadaceae*—a family known for degrading complex organic matter [57]—but this functional group was not detected in the AP group until day 90.

### 4.3. P. putida Affected the Microbial Functional in the Casing Soil

Actinomycetes mainly release nutrients by degrading recalcitrant organic matter and produce antibacterial compounds to inhibit potential competitors and pathogenic bacteria, thereby providing a suitable living environment for morels [58]. Meanwhile, the bacillota phylum plays the typical role of plant rhizosphere beneficial bacteria, possibly by dissolving nutrients (such as phosphorus and potassium) and producing plant hormones to stimulate the growth of fungal hyphae [59,60]. The delayed appearance of *Sphingomonadaceae* suggests that inoculation with *P. putida* may have temporarily inhibited the colonization of certain functional microorganisms. Critically, this temporal shift in microbial succession has potential functional consequences for *M. sextelata* nutrition. Sphingomonadaceae are specialists in breaking down complex aromatic compounds and recalcitrant organic matter. Their delayed proliferation in the inoculated group likely postpones the mineralization of these complex carbon sources, potentially synchronizing the release of soluble nutrients with the later stages of primordia formation and fructification in morels. This altered timing of nutrient availability could be a key mechanism behind the observed promotion of morel growth, ensuring that a pulse of nutrients is available during the critical reproductive phase.

A noteworthy comparison can be drawn with the cultivation of *A. bisporus*, which, like *Morchella*, exhibits a strong dependence on casing soil for fructification, without which commercial yields are unattainable [61]. However, a fundamental distinction lies in their nutritional strategies and cultivation timelines. The substrate for *A. bisporus* undergoes pre-composting, a process that pre-degrades complex organic matter, creating a readily available nutrient pool [62]. This allows for a short and intensive cropping cycle (approximately 30 days), within which a single inoculation of *P. putida* might be sufficient to exert its beneficial effects throughout the production period. In contrast, *M. sextelata* employs a distinct nutritional strategy, a saprotrophic phase supported by an external nutrient bag during mycelial growth, followed by the transition to a low-nutrient requirement for primordia formation. This extended life cycle, often spanning up to 90 days, presents a different ecological challenge. The transient nature of the inoculated *P. putida* population observed in our study suggests that a single inoculation may be insufficient to persist and exert influence across the entire prolonged cultivation period. This biological and operational dichotomy underscores that microbial management strategies successful in one system cannot be directly transposed to another without considering the intrinsic crop physiology and cultivation framework.

Bacteroidetes and Pseudomonadota are capable of rapidly degrading organic matter [63,64], thereby enhancing soil nutrient availability and significantly promoting the early establishment and growth of morels. However, this accelerated degradation may pose a risk of depleting soil fertility in later stages, potentially impairing morel development (Figure 3 and Figure 5C). To counteract these potential drawbacks in real-world agricultural practice, we propose integrating *P. putida* inoculation with complementary soil health management strategies. These include (1) the application of slow-decomposing organic amendments (e.g., biochar or lignin-rich compost) after harvesting to replenish soil organic carbon and buffer against fertility loss; and (2) the practice of crop rotation with plants (in southern China, rice is often chosen [65]) to disrupt pathogen cycles and allow for the recovery of a diverse and suppressive native microbiota. *Streptomyces*, as producers of various natural antibiotics, may contribute to soil disease suppression [66]. A decline in their relative abundance could thus weaken the soil’s overall capacity to resist pathogenic microbes. Some studies report that *Pseudomonas* species secrete antibiotic compounds, with *fluorescent Pseudomonads* producing phenazine antibiotics that inhibit Fusarium wilt [67], and antagonize mycotoxin-producing fungi like *Alternaria* and *Fusarium* in wheat [68]. This antimicrobial activity may explain the reduced abundance of *Streptomyces* observed in the AP group compared to the CK group.

This introduces an important ecological trade-off. The production of phenazines by the inoculated *P. putida* likely provides a potent, specific defense against certain fungal pathogens, creating a competitive advantage for itself and other antibiotic-resistant bacteria. However, this comes at the cost of reducing the phylogenetic and functional diversity of the native antibiotic-producing community, as evidenced by the decline in *Streptomyces*. While phenazines are effective, a diverse community of Actinobacteria produces a vast arsenal of novel antibiotics, which constitutes a more robust, “insurance” strategy against a wider range of potential pathogens. Thus, the inoculation strategy effectively exchanges a broad-spectrum, native defense system for a more targeted, introduced one, a trade-off that must be considered in long-term disease management. While this study focused on a single cultivation cycle, the long-term consequences of such a reduction could be profound. Over successive crops, a diminished native suppressive microbiome might allow for soil-borne pathogens (e.g., *Lecanicillium* and *Mycogone*) to accumulate, potentially leading to increased disease incidence and a consequent decline in *Morchella* yield and overall crop health. This risk underscores the importance of monitoring soil microbiome health in continuous cultivation systems

### 4.4. Effects of Pseudomonas putida on Soil Functional Genes and the ACC Metabolic Pathway

Bacteria of the genus *Pseudomonas* are known to act as biological control agents against soil-borne diseases, thereby enhancing soil and plant health [69]. Harifazizi et al. isolated the *fluorescent Pseudomonas* strains Ps170 and Ps117, whose nitrofurantoin and pyrrolidone derivatives exhibited significant efficacy in controlling fire blight caused by *Erwinia amylovora* [70]. Yang also reported that inoculation with *Pseudomonas chlororaphis* alleviated soil-borne diseases caused by *Paecilomyces penicillatus* [71]. For the soil microbial community, inoculation with *P*. *putida* represents an invasion by an exogenous species. The biological control function of *P*. *putida* and the potentially excessive inoculation dose may explain this phenomenon. Subsequent experiments will further explore the optimal dose of *P*. *putida*. As early as 1979, Adams and Yang identified 1-aminocyclopropane-1-carboxylic acid (ACC) as the direct precursor of ethylene biosynthesis [72]. In this pathway, methionine is first converted to S-adenosylmethionine (SAM) by SAM synthase. ACC synthase then catalyzes the formation of ACC from SAM, and ACC oxidase subsequently converts ACC into ethylene. Zhang et al. reported an ACC-dependent ethylene synthesis pathway in *A. bisporus* identical to that in higher plants [73]. In this study, we detected ACC accumulation in the soil both before and after morel mushroom cultivation (Figure 2A), suggesting that morels may possess the same ethylene synthesis pathway as *A. bisporus*. We therefore compared the abundance of related enzymes across experimental groups (Figure 8B). This pattern positively correlated with the abundance trend of *Pseudomonas* in microbial communities (Figure 8E). Given that *Pseudomonas* putida KT2440 can utilize ACC, these results indicate that the abundance of ACC deaminase genes is closely linked to the presence of *Pseudomonas*. Based on the observed ACC accumulation in morels, we propose that supplementary inoculation with *P. putida* during primordia formation may help alleviate ethylene-mediated inhibition of morel growth.

## 5. Conclusions

This study systematically investigated the effects of *Pseudomonas putida* inoculation on the microbial community structure and function in the casing soil used for Morchella cultivation. The results indicated that while *P. putida* inoculation promoted the growth of Morchella, it also accelerated the depletion of soil available nitrogen, phosphorus, and potassium, which may lead to insufficient soil fertility in the later stages of cultivation, and thereby decreasing the yield of Morchella. In addition, the inoculation enhanced bacterial diversity and microbial community stability and increased the relative abundance of Morchella, but reduced the abundance of the biocontrol bacterium Streptomyces. A positive correlation was also observed between *P. putida* and soil ACC deaminase activity, suggesting its potential role in promoting Morchella growth by degrading ACC and reducing the ethylene levels. These findings highlight the positive effects of *P. putida* on Morchella growth through the regulation of soil micro-environment and microbial community. However, as this study focused on short-term growth-promoting effects, the potential long-term impacts of its application on soil micro-ecology remain unclear. Therefore, future research should first elucidate the specific mechanisms by which *P. putida* enhances Morchella growth. Based on these mechanisms, specialized microbial inoculants centered on this strain can be developed, and their efficacy in improving Morchella yield and mitigating continuous cropping obstacles should be verified through field trials, which will provide new strategies and theoretical foundations for the use of growth-promoting bacteria in edible fungus cultivation.

## Data Availability

The raw metagenomic data reported in this paper have been deposited in the NCBI Sequence Read Archive (SRA) under the BioProject accession number PRJNA1311151 (accessed on 2 September 2025).

## Supplementary Materials

The following supporting information can be downloaded at: https://www.mdpi.com/article/10.3390/jof11110775/s1, Table S1: α diversity indices of bacteria and fungi at different stages in the CK and AP groups. Table S2: RDA analysis comparison table for Bacteria and Fungi. Figure S1: ACC content chromatogram. (A–C) Chromatogram of the CK group. (D–F) Chromatogram of the AP group. RT, retention time; MA, peak area; MH, peak height; SN, Signal-to-Noise Ratio. Figure S2: KEGG pathway enrichment analysis in carbon, phosphorus and nitrogen cycle. (A) carbon cycle. (B) phosphorus cycle. (C) nitrogen cycle. Microbial gene functions were changed after inoculating *P. putida*. Blue, enriched in CK group; red, enriched in AP group. Asterisk denotes reporter score of pathways > 1.65 or  <−1.65.
