# Supplementary material for: The Effect of Pseudomonas putida on the Microbial Community in Casing Soil for the Cultivation of Morchella sextelata
Source: J Fungi (Basel). 2025 Oct 27;11(11):775. doi: 10.3390/jof11110775 (PMC12653408; doi:10.3390/jof11110775)
Supplement: Supplementary file 1 [file jof-11-00775-s001.zip › jof-3890072-supplementary.pdf]

**Table S1.** $\alpha$ diversity indices of bacteria and fungi at different stages in the CK and AP groups

| Days | Bacteria CK Group | | Bacteria AP Group | | Fungi CK Group | | Fungi AP Group | |
|---|---|---|---|---|---|---|---|---|
| | Chao index | Shannon index | Chao index | Shannon index | Chao index | Shannon index | Chao index | Shannon index |
| 0 d | 2575.7 ±67.2c | 2.8 ±0.2b | 2495.3 ±82.6c | 2.7 ±0.1c | 210.3 ±9.3b | 3.1 ±0.1a | 151.7 ±37.9b | 2.8 ±0.2a |
| 30 d | 3496.3 ±169.2b | 4.1 ±0.3a | 3381.0 ±110.5b | 4.1 ±0.1ab | 802.3 ±8.4a | 1.5 ±0.4b | 812.0 ±49.6a | 1.2 ±0.5b |
| 60 d | 3546.0 ±47.6b | 4.1 ±0.2a | 3382.7 ±84.7b | 3.9 ±0.2b | 838.3 ±21.1a | 1.2 ±0.3b | 846.3 ±34.4a | 1.6 ±0.6b |
| 90 d | 3913.0 ±30.8a | 4.4 ±0.1a | 3877.7 ±46.2a | 4.4 ±0.1a | 821.3 ±9.6a | 2.0 ±0.3b | 874.7 ±26.3a | 2.0 ±0.1ab |

Notes: Values are mean values ± SE (n = 3). Values within the same column followed by different letters indicate significant differences ($p < 0.05$).

**Table S2.** RDA analysis comparison table for Bacteria and Fungi

**Bacteria**

| Soil Properties | Pseudomonas | Streptomyces | Flavobacterium | Bradyrhizobium | Pedobacter | Rhizomicrobum | Asticcacaums | Sphingomonas | Rhodanobacter | Mucilaginibacter |
|---|---|---|---|---|---|---|---|---|---|---|
| OC | + | + | - | - | + | - | - | - | - | + |
| TN | - | - | - | + | o | + | + | + | + | + |
| TP | + | o | + | + | - | + | + | + | + | - |
| TK | + | o | + | + | - | + | + | + | + | - |
| AN | + | - | + | + | - | + | + | + | + | - |
| AvP | + | + | + | + | - | + | + | o | o | - |
| AK | + | + | + | - | - | - | - | - | - | - |

**Fungi**

| Soil Properties | Pseudogymnoascus | Morchella | Sugiyamaella | Monilinia | Fusarium | Aspergillus | Penicillim | Paecilomyces | Coniochaeta | Spathaspora |
|---|---|---|---|---|---|---|---|---|---|---|
| OC | - | + | + | + | + | + | + | + | + | + |
| TN | o | - | - | - | - | - | - | - | - | - |
| TP | + | + | + | + | + | + | + | + | - | - |
| TK | + | - | - | - | - | - | - | - | - | - |
| AN | + | - | - | - | - | - | - | - | - | - |
| AvP | - | - | - | - | - | - | - | - | o | + |

| | | | | | | | | | | |
|---|---|---|---|---|---|---|---|---|---|---|
| AK | + | - | - | - | - | - | - | - | - | - |

Notes: + indicates a positive correlation, - indicates a negative correlation, o indicates little to no Correlation.

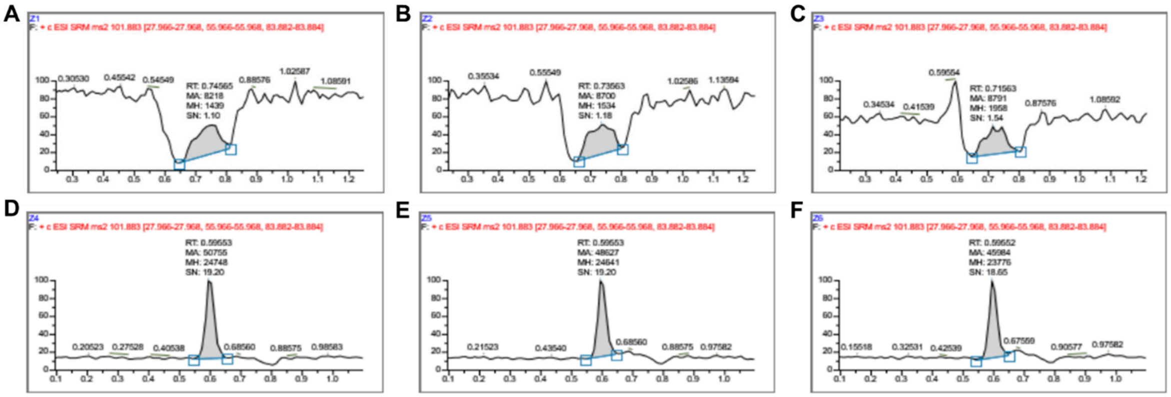


**FigureS1**. ACC content chromatogram. (A-C) Chromatogram of the CK group. (D-F) Chromatogram of the AP group. RT, retention time; MA, peak area; MH, peak height; SN, Signal-to-Noise Ratio.

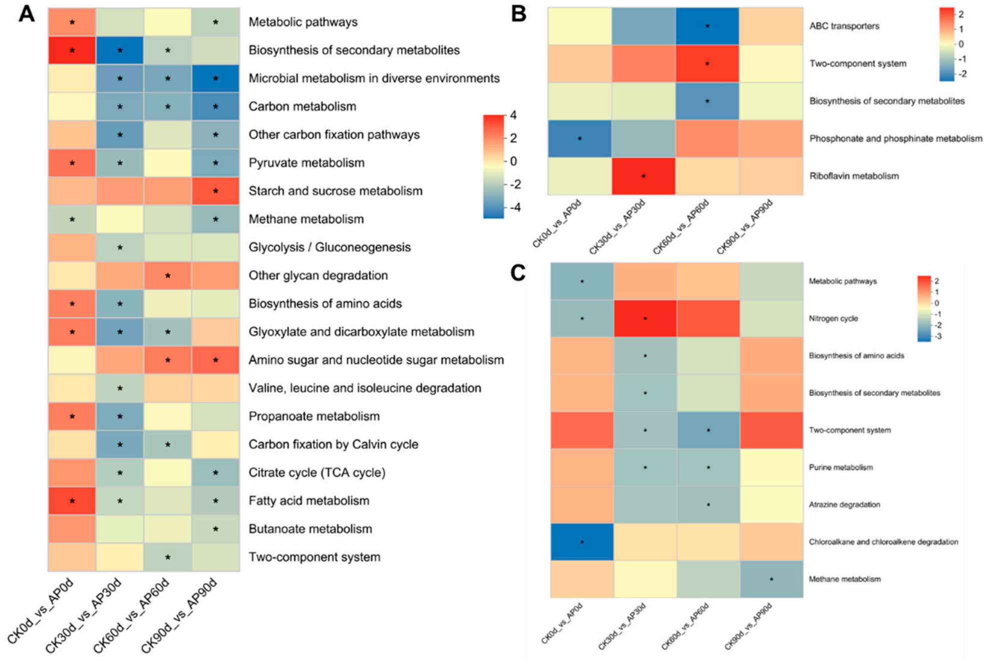


**FigureS2.** KEGG pathway enrichment analysis in carbon, phosphorus and nitrogen cycle. (A) carbon cycle. (B) phosphorus cycle. (C) nitrogen cycle. Microbial gene functions were changed after inoculating P. putida. Blue, enriched in CK group; red, enriched in AP group. Asterisk denotes reporter score of pathways > 1.65 or < −1.65.
